# A large mid-Holocene estuary was not present in the lower River Murray, Australia

**DOI:** 10.1038/s41598-021-90025-9

**Published:** 2021-06-08

**Authors:** J. Tibby, B. Bourman, C. Wilson, L. M. Mosley, A. P. Belperio, D. D. Ryan, P. A. Hesp, C. V. Murray-Wallace, G. Miot da Silva, S. R. Dillenburg, D. Haynes

**Affiliations:** 1 Department of Geography, Environment and Population, University of Adelaide, Adelaide, 5005 Australia; 2 Sprigg Geobiology Centre, University of Adelaide, Adelaide, 5005 Australia; 3 School of Earth, Atmospheric and Life Sciences, Faculty of Science, Medicine and Health, University of Wollongong, Wollongong, NSW 2522 Australia; 4 College of Science and Engineering, Flinders University, Bedford Park, 5042 Australia; 5 College of Humanities Arts and Social Sciences, Flinders University, Beford Park, South 5042 Australia; 6 Centre for Australian Biodiversity and Heritage (CABAH), Adelaide, Australia; 7 School of Biological Sciences, University of Adelaide, Adelaide, 5005 Australia; 8 Minotaur Exploration, Norwood, 5067 Australia; 9 MARUM, University of Bremen, Leobener Str. 8, 28359 Bremen, Germany; 10 Beach and Dune Systems (BEADS) Laboratory, College of Science and Engineering, Flinders University, Bedford Park, 5042 Australia; 11 School of Earth, Atmospheric and Life Sciences, University of Wollongong, Wollongong, NSW 2522 Australia; 12 Geosciences Institute, Federal University of Rio Grande do Sul, Porto Alegre, 91509-900 Brazil; 13 School of Physical Sciences, University of Adelaide, Adelaide, 5005 Australia

**Keywords:** Limnology, Environmental monitoring

**arising from**: A. M. Helfensdorfer et al.; *Scientific Reports* https://doi.org/10.1038/s41598-019-39516-4 (2019).

**arising from**: A. M. Helfensdorfer et al.; *Scientific Reports* https://doi.org/10.1038/s41598-020-61800-x (2020).

## Introduction

Recent research has suggested that during the mid-Holocene (c. 8500 to 5000 cal yr BP) a large estuary occupied the lower River Murray and its terminal lakes (Lakes Alexandrina and Albert: herein the Lower Lakes) in South Australia. This research has questioned both reconstructions of past River Murray discharge and contemporary environmental water provisions aimed at maintaining the freshwater state of the Lower Lakes. We show that (1) a large mid-Holocene estuary extending into the lower River Murray was not physically possible, and (2) that the River Murray and Lower Lakes were predominantly fresh during the mid-Holocene. Sea level was well below present at the time of purported initiation of estuarine sedimentation and, therefore, could not have allowed formation of an estuary. Holocene human occupation of the lower River Murray valley, that was reliant on freshwater resources, negates the existence of a large estuary in the valley. A variety of freshwater indicators in sediments from in, and around, the Lower Lakes negate the notion of significant marine incursion. Hence, current management of the Lower Lakes as freshwater ecosystems is consistent with their Holocene history.

### Inferred history of the lower River Murray

**Figure 1:**
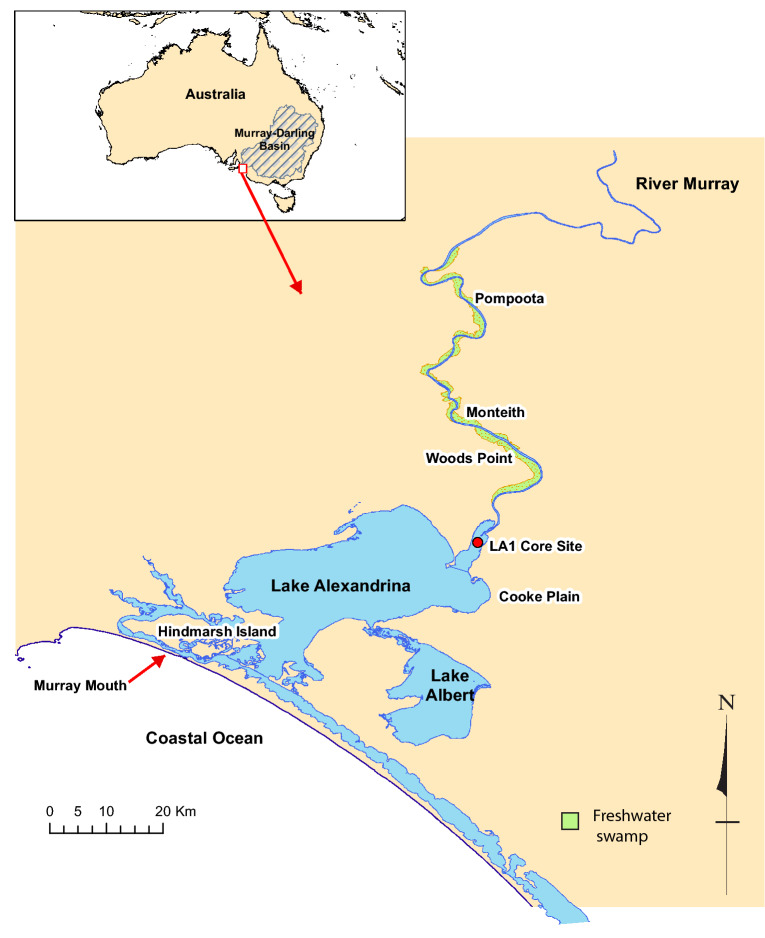
The lower River Murray and sites mentioned in the text. Monteith is the location of core and cone penetrometer research reported in^2^.

Helfensdorfer et al.^1,2^ proposed that a + 2 m AHD sea level highstand 7000–6000 year BP resulted in marine incursion into the Lower Lakes, forced the estuarine limit > 200 km upstream and generated a central basin, which captured a mud deposit up to 3 km wide, > 60 km long and > 10 m thick, dated to 8518–5067 cal year BP^2^ at the floodplain site of Monteith^2^ (Fig. 1). Furthermore, a shift to freshwater conditions was proposed as a result of sea level falling to its present position after 4 ka BP^1,2^. It was further claimed that this “sediment trap”^2^ prevented sediment delivery to the ocean, requiring a re-evaluation of south-eastern Australian climate reconstructions from marine sediments off the River Murray mouth^3^; a series of propositions we reject.

### Evidence of freshwater dominance in the lower River Murray and Lower Lakes in the mid-Holocene

**Figure 2:**
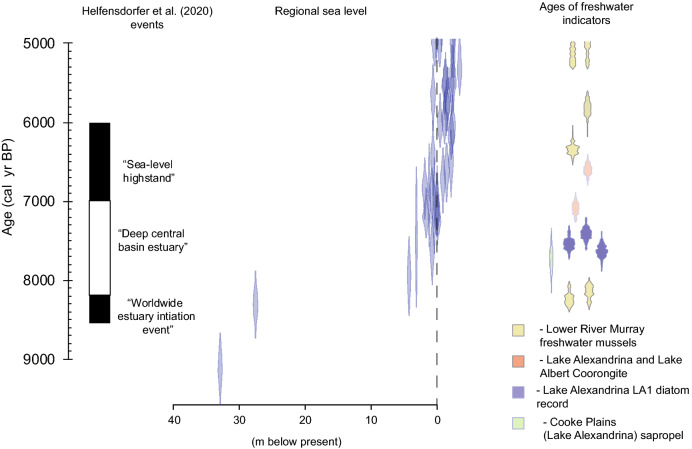
History of the lower River Murray inferred in^1,2^, South Australian high quality sea level data^11^, ages for freshwater mussels from the lower River Murray region and dates on a number of freshwater indicators from Lakes Alexandrina and Albert including Coorongite—a freshwater-derived sediment, dates for freshwater sapropel units and diatom record LA1^20^ which is dominated by freshwater diatoms^20^. All ages in this plot were recalibrated for this study, with the marine ages (ref^11^) calibrated with a ΔR of 84 years and a standard deviation of 59 years derived from the 3 most proximal sites in the CALIB marine reservoir correction data based (http://calib.org/marine/). Note that for the LA1 record we have assumed that the ages represent the time of deposition and did not gradually accumulate over the past c. 7000 years as suggested in the original research.

There is strong evidence freshwater dominated the Lower Lakes in the mid-Holocene (Fig. 2). A sapropel unit underlying the Cooke Plains, a now-exposed former embayment of Lake Alexandrina, dated to 6930 ± 150 year BP originates from freshwater which was deep enough to stratify^4^. This unit is associated with expansion of Lake Alexandrina during a mid-Holocene humid period^3^. Sub-fossil diatoms in the Lower Lakes also indicate the dominance of freshwater at this time (Fig. 2^5^) as does freshwater algae-derived Coorongite deposited in Lake Albert and Lake Alexandrina (Fig. 2^6^).

Archaeological investigations of freshwater shell midden sites (dominated by *Velesunio ambiguus*) between Pompoota and Woods Point (~ 86 km upstream of the Murray Mouth) demonstrate a continuity of human occupation and reliance on freshwater from c. 8400 year BP to the time of European occupation^7^. These sites include the Swanport site < 7 km upstream of Monteith which was within the hypothesised estuary extent outlined in^2^ (Fig. 1). Freshwater mussels have limited tolerance of brackish conditions. Adult freshwater mussels and their larvae tolerate saline water for only short periods of time, but populations generally do not persist in salinities above about 3.5 g L^−1^^18^. This renders the proposed presence of persistent brackish conditions (see Fig. 2a in^2^) in the lower River Murray improbable.

### Regional sea level history and the lower River Murray

A combination of eustatic and relative sea level evidence is used to support the notion of an estuary penetrating into the lower River Murray from 8.5 ka BP^2^. Subsequent hypothesised freshening^2^ is argued to result predominantly from a fall in sea level rather than changing inflows^1^. Given this, and the very low gradient environment of the study area, accurate characterisation of the sea level history is critical. Helfensdorfer et al. argued that a River Murray estuary began forming 8500 year BP following a “worldwide” pattern of estuary initiation in response to a “step wise” jump in sea level^2^. However, relative sea levels, even in far field basins^9^, including around Australia^10^ varied by tens of metres at this time. Hence the notion of global estuary initiation is neither correct nor relevant. Rather, it is relative regional sea level that is pertinent to understanding the evolution of the lower River Murray. The southern Australian coast is affected by spatially variable neotectonism and glacio-hydro-isostatic adjustments^11^. Critically, at the time of hypothesised estuary commencement, regional sea level was > 20 m lower than at present^11,12^ (Fig. 2). Indeed, even the global eustatic record indicates sea level was > 15 m below present at this time^12^ with the notion of a step wise sea level increase rejected^12^. Importantly, the local sea level record used in^2^ is not the optimal curve from the original study (Fig. 3^11^) since, as noted in that study, it contains indicators that overestimate sea level (i.e. seagrass facies, beach ridges and cheniers)^11^. Additionally, regional sea level data from^11^ are plotted in ^14^C years, while the global data are presented in calibrated years (Fig. 6 in^2^).

Helfensdorfer et al.^1,2^ provide no direct evidence for a mid-Holocene + 2 m AHD sea level in the vicinity of the Murray Mouth. Instead a large sandflat at ~  + 2 m AHD on the southern half of Hindmarsh Island near the Murray Mouth^13^ is mentioned. Sand flats are not reliable sea-level indicators; they aggrade above mean sea level as king tides and storm surges flatten sand drift trapped by salt-marsh vegetation. The sand flat actually varies in elevation between 1 and 2 m and is sporadically underlain by mid-Holocene shells (including *Tellinota albinella, Macomana deltoidalis* and *Hiatula biradiata*) 0.6 m to 1.0 m below the surface. A radiocarbon age of 5980 + 80 years BP (WK 4784) was obtained on shell from beneath the sand flat demonstrating a mid-Holocene sea level of ~  + 1 m, one half of that proposed by Helfensdorfer et al.^1,2^.

On Hindmarsh Island, last interglacial (LIG; c. 125 ka BP) and Holocene shoreline successions (c. 6000 year BP) broadly coincide. LIG sea level in southern Australia was slightly higher than + 2 m AHD^14^. However, within the Murray Estuary, the combination of local basin subsidence, hydro-isostasy, ocean siphoning and a minor contribution from near-field glacio-isostasy have affected the elevations of the LIG and Holocene shorelines, explaining their local coincidence. Given the more proximal location of the Murray Mouth to the edge of the Lacepede Shelf, a value of + 1 m AHD is more realistic for relative sea level for the mid-Holocene highstand, as originally noted in^4^ and is consistent with other proximal sites, such as Port Lincoln^8^ and The Granites at the SE end of the Younghusband Peninsula^15^. The river valleys of nearby Fleurieu Peninsula have mid-Holocene alluvium grading to a sea level at ~  + 1 m AHD. This is the most reliable proximate estimate for mid-Holocene relative sea level at the Murray Mouth^16^, with similar estimates for sea level at The Granites once the effect of subsequent tectonism is removed^15^.

### Assessment of Helfensdorfer et al. (2019, 2020)

Modelling is used to infer marine conditions in the lower River Murray^1,2^. This modelling is unrealistic as (1) it uses a combination of unjustifiable sea level highstand (+ 2 m AHD) and width of the Murray Mouth in several scenarios in the model setup (orders of magnitude greater than current mouth width of a few hundred metres^5,12^); (2) Millennium Drought salinity levels (the driest period in the > 100 year record) are used as the initial condition for the model. These are likely to be elevated relative to the mid-Holocene river and Lower Lakes salinity due to additional factors including river regulation and land salinisation^17^; and (3) a model which ran for only 20 days and assumed it had reached steady state. Given the long distances from inflow to the Murray Mouth, the low velocities modelled^2^ and storage volumes in the model domain, the simulation period is far too short for salinity to reach steady state. In reality, it took several months (Lake Alexandrina) to years (Lake Albert) for salinities to recover from the Millennium Drought^18^.

To summarise, the multiple lines of physical evidence for freshwater in the lower River Murray and Lower Lakes are diametrically opposed to the inferred significant marine incursion modelled by Helfensdorfer et al.^1,2^. Furthermore, no definitive evidence exists for estuarine deposition of Holocene clays in the Murray Gorge. It was argued that the fine-grained sediments are not ‘normal’ riverine deposits^2^. However, it is ‘normal’ for slow flowing, extremely low gradient, lowland rivers to transport clays and silts as suspended load^3^. Moreover, sediments to depths of 3 m on floodplains upstream and downstream of Monteith are indicative of fluvial deposition, because they are composed of quartz, kaolin, illite and smectite, with traces of feldspars^19^. No marine or estuarine biota (e.g. molluscs, dinoflagellates or diatoms) is reported from the clays at Monteith^1,2^. While it was suggested^1,2^ that higher salinities could have aided clay flocculation and deposition^2^, this process can happen in slow moving freshwater, while dense reeds beds may have aided sediment trapping. Indeed, the deposition of clays before, and after the inferred mid-Holocene estuary^2^ demonstrates that this can occur in the absence of elevated salinity. Rather, and in conclusion, we posit that the “estuary” clays were deposited by rapid fluvial aggradation of fine-grained sediments between 10,000 and 7000 year BP. The multiple lines of evidence show that the Lower Lakes and lower River Murray were predominantly freshwater and claims that they were marine and estuarine, respectively, in the mid-Holocene are unfounded.

## References

[1] Helfensdorfer AM, Power HE, Hubble TCT (2019). Modelling Holocene analogues of coastal plain estuaries reveals the magnitude of sea-level threat. Sci. Rep..

[2] Helfensdorfer AM, Power HE, Hubble TCT (2020). Atypical responses of a large catchment river to the Holocene sea-level highstand: The Murray River, Australia. Sci. Rep..

[3] Gingele F, De Deckker P, Norman M (2007). Late Pleistocene and Holocene climate of SE Australia reconstructed from dust and river loads deposited offshore the River Murray Mouth. Earth Planet. Sci. Lett..

[4] von der Borch C, Altmann M (1979). Holocene stratigraphy and evolution of the Cooke Plains Embayment, a former extension of Lake Alexandrina, South Australia. Trans. R. Soc. South Aust..

[5] Tibby J, Haynes D, Muller K (2020). The predominantly fresh history of Lake Alexandrina, South Australia, and its implications for the Murray-Darling Basin Plan: A comment on Gell (2020). Pac. Conserv. Biol..

[6] Fitzpatrick, R. W., Shand, P. & Mosley, L. M. In *Natural History of the Coorong, Lower Lakes and Murray Mouth Region (Yarluwar-Ruwe)* (eds L. Mosley *et al.*) 227–252 (University of Adelaide Press, 2018).

[7] Wilson C, Fallon S, Trevorrow T (2012). New radiocarbon ages for the Lower Murray River, South Australia. Arch Oceania.

[8] Walker KF (2017). Reproductive phenology of river and lake populations of freshwater mussels (Unionida: Hyriidae) in the River Murray. Molluscan Res..

[9] Mann T (2019). Holocene sea levels in Southeast Asia, Maldives, India and Sri Lanka: The SEAMIS database. Quat. Sci. Rev..

[10] Lewis SE, Sloss CR, Murray-Wallace CV, Woodroffe CD, Smithers SG (2013). Post-glacial sea-level changes around the Australian margin: A review. Quat. Sci. Rev..

[11] Belperio AP, Harvey N, Bourman RP (2002). Spatial and temporal variability in the Holocene sea-level record of the South Australian coastline. Sed. Geol..

[12] Lambeck K, Rouby H, Purcell A, Sun Y, Sambridge M (2014). Sea level and global ice volumes from the Last Glacial Maximum to the Holocene. PNAS.

[13] Bourman RP, Murray-Wallace CV, Belperio AP, Harvey N (2000). Rapid coastal geomorphic change in the River Murray Estuary of Australia. Mar. Geol..

[14] Murray-Wallace CV (2016). Last interglacial (MIS 5e) sea-level determined from a tectonically stable, far-field location, Eyre Peninsula, southern Australia. Aust. J. Earth Sci..

[15] Dillenburg, S. R. *et al.* Geochronology and evolution of a complex barrier, Younghusband Peninsula, South Australia. *Geomorphology* **354**, 107044 (2020).

[16] Bourman RP (2006). River terraces of the Fleurieu Peninsula. South Aust. Geogr. J..

[17] Mosley LM (2012). The impact of extreme low flows on the water quality of the Lower Murray River and Lakes (South Australia). Water Res. Manag..

[18] Aldridge, K., Mosley, L. M. & Oliver, R. In *Natural History of the Coorong, Lower Lakes and Murray Mouth Region (Yarluwar-Ruwe)* (eds L. Mosley *et al.*) 253–270 (University of Adelaide Press, 2018).

[19] Fitzpatrick RW, Shand P, Mosley LM (2017). Acid sulfate soil evolution models and pedogenic pathways during drought and reflooding cycles in irrigated areas and adjacent natural wetlands. Geoderma.

[20] Fluin J, Gell P, Haynes D, Tibby J, Hancock G (2007). Palaeolimnological evidence for the independent evolution of neighbouring terminal lakes, the Murray Darling Basin, Australia. Hydrobiologia.

